# Assessment of Conventionally and Digitally Fabricated Complete Dentures: A Comprehensive Review

**DOI:** 10.3390/ma15113868

**Published:** 2022-05-28

**Authors:** Manal Q. Mubaraki, Mohammed M. Al Moaleem, Abdulrahman H. Alzahrani, Mansoor Shariff, Saeed M. Alqahtani, Amit Porwal, Fuad A. Al-Sanabani, Shilpa Bhandi, João Paulo Mendes Tribst, Artak Heboyan, Shankargouda Patil

**Affiliations:** 1 Saudi Board Prosthodontic PGY3, College of Dentistry, King Khalid University, Abha 62529, Saudi Arabia; manalgm1994@gmail.com; 2 Department of Prosthetic Dental Science, College of Dentistry, Jazan University, Jazan 45142, Saudi Arabia; aporwal2000@gmail.com (A.P.); fualsanabani@jazanu.edu.sa (F.A.A.-S.); 3 Faculty of Dentistry, University of Ibn al-Nafis for Medical Sciences, Sana’a 4337, Yemen; 4 Faculty of Dentistry, Taif University, Taif 21944, Saudi Arabia; dr.a.alzhrani@tudent.edu.sa; 5 Prosthetic Department, College of Dentistry, King Khalid University, Abha 62529, Saudi Arabia; mansoor_shari_@hotmail.com (M.S.); smaalqahtani@kku.edu.sa (S.M.A.); 6 Department of Restorative Dental Sciences, Division of Operative Dentistry, College of Dentistry, Jazan University, Jazan 45142, Saudi Arabia; shilpa.bhandi@gmail.com; 7 Department of Dental Materials, Academic Centre for Dentistry Amsterdam (ACTA), University of Amsterdam and Vrije Universiteit Amsterdam, 1081 LA Amsterdam, The Netherlands; j.p.mendes.tribst@acta.nl; 8 Department of Prosthodontics, Faculty of Stomatology, Yerevan State Medical University after Mkhitar Heratsi, Str. Koryun 2, Yerevan 0025, Armenia; heboyan.artak@gmail.com; 9 Department of Maxillofacial Surgery & Diagnostic Sciences, Division of Oral Pathology, College of Dentistry, Jazan University, Jazan 45142, Saudi Arabia

**Keywords:** computer-engineered complete denture, digital complete denture, polymers, CAD/CAM complete denture, denture base material

## Abstract

CAD/CAM technology is gaining popularity and replacing archaic conventional procedures for fabricating dentures. CAD/CAM supports using a digital workflow reduce the number of visits, chair time, and laboratory time, making it attractive to patients. This study aimed to provide a comparative review of complete dentures manufactured using CAD/CAM and conventional methods. The PubMed/Medline, Science Direct, Cochrane, and Google Scholar databases were searched for studies published in English within the last 11 years (from 2011 to 2021). The keywords used were “computer-engineered complete dentures”, “CAD/CAM complete dentures”, “computer-aided engineering complete dentures”, and “digital complete dentures”. The search yielded 102 articles. Eighteen relevant articles were included in this review. Overall, computer-engineered complete dentures have several advantages over conventional dentures. Patients reported greater satisfaction with computer-engineered complete dentures (CECDs) due to better fit, reduced chair time, shorter appointments, and fewer post-insertion visits. CAD/CAM allows for precision and reproducibility with fewer procedures compared to conventional dentures. Polymethyl methacrylate is used as the denture base material for conventional dentures. For CECDs, the resin can be modified and cross-linked to improve its mechanical properties. The advantages of CECDs include a reduced number of appointments, saving chairside time, a digital workflow allowing easy reproducibility and greater patient satisfaction with a better fit.

## 1. Introduction

Complete dentures are removable dental prostheses that rehabilitate the whole dentition as well as the related structures of the maxilla and/or mandible [1]. According to the Glossary of Digital Dental Terms, a digital denture is a complete prosthesis that is formed by or through automation using CAD/CAM (computer-aided design and computer-aided manufacturing) and computer-aided engineering (CAE) [2]. CAE refers to the field of engineering where data are gathered and investigated before being applied to design procedures, including in the manufacturing methods for biomedical prostheses [3,4]. Conventional complete dentures (CDs) are the most commonly used prosthetic modality to rehabilitate edentulous patients. They have drawbacks such as requiring multiple visits and high laboratory expenses. The denture base of CDs may not have an intimate fit with the underlying tissues due to polymerization shrinkage of the acrylic resin. Creating a duplicate complete denture is a cumbersome process [5,6,7]. Traditional conventional complete dentures remain a valuable solution. However, newer treatments such as “all on four” rehabilitation are gaining popularity [8].

The implementation of CAD/CAM systems in dentistry four decades ago ushered in a new age for the successful fabrication of full-coverage crowns, fixed dental prostheses, and superstructures for natural teeth as well as dental implants [5,9,10]. Complete dentures manufactured using CAD/CAM represents a new era in removable prosthodontics. Numerous commercial CAD/CAM systems are available for the design and manufacture of CDs [5,6,11]. They allow customization of tooth set-up, verification of preceding steps before the trial appointment, and the ability to design a product that is clinically predictable [7,12,13]. CAD/CAM systems allow for improvements in both the mechanical and surface properties [14].

**Figure 1:**
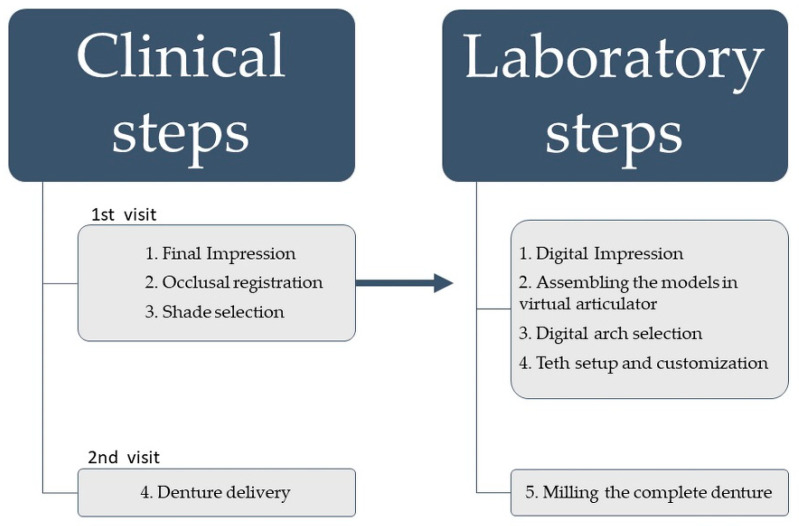
Workflow of CECDs.

Dentists and prosthodontists are believed to be familiar with the workflow of the clinical and laboratory steps required for conventional denture fabrication. For the purposes of this article, Table 1 presents the details of case reports on the use of CECDs (computer-engineered complete dentures) technology that have been published in different countries [10,12,14,15,16,17,18,19,20,21,22,23,24,25]. The table includes the names of the authors, the year of the case report’s publication, the country in which the case study was carried out, the number of visits, the patients’ gender, the age of the patient, the type of edentulous arches, the technique used, conclusions, and positive points. Figure 1 shows the workflow of manufacturing CECDs. 

This study aimed to provide a comparative analysis of complete dentures fabricated by both digital and conventional methods. We outline the techniques and materials used for the fabrication of digital and conventional complete dentures and highlight their advantages and disadvantages.

## 2. Literature Review

### 2.1. Data Collection

The PubMed/Medline, Science Direct, Cochrane, and Google Scholar databases were searched for literature published in English within the last 11 years (from 2011 to 2021). The keywords used were “computer engineered complete denture”, “CAD/CAM complete dentures” “computer-aided engineering complete dentures”, and “digital complete dentures”. The inclusion criteria were studies carried out in clinics, case reports or series, and reviews. The search strategy included revising the titles and abstracts to select articles that met the inclusion criteria and exclude those that did not. Only papers published in the English language were reviewed (A. P. and M. Al M) by reading the title and abstract. The contents of each paper were then summarized. A researcher evaluated the validity of the studies and identified duplications. Two investigators (Al M.M and M.Y) read all of the titles and abstracts individually and carefully evaluated them. The researchers had to agree as to whether each study was relevant. Finally, 18 full-text articles were selected and analyzed. Interventional studies involving animals or humans and other studies requiring ethical approval had to contain the approval information and the corresponding ethical approval code. Didactically, the present review was divided into subtopics presented below.

### 2.2. History of CAD/CAM

The first digitally made removable complete denture was generated using 3D printing technology by Maeda et al. [26]. In 1997, Kawahata et al. [27] used a wax block with computerized arithmetic regulator milling technology. Later on, Busch et al. [28] described a digital tooth arrangement based on anatomic dimensions and averages. Kattadiyil et al. produced CAD software that processed an automatic tooth setup, semiautomatic aesthetic scheming, individualized gingival contouring, and base plate establishment [29].

Kanazawa et al. used cone-beam CT scanning combined with either a rapid prototyping method or a milling method to fabricate computer-engineered complete dentures (CECDs) [24]. In 2012, Goodacre et al. recommended the use of recorded intaglio and the cameo surfaces of CD denture bases with the areas where teeth were located [9]. The first denture base was milled from polymethyl methacrylate (PMMA) to which denture teeth were bonded by hand and placed in the patient’s mouth.

### 2.3. Manufacturing of Computer-Engineered Complete Dentures

Computer-engineered complete dentures (CECDs) can be manufactured in two ways: The first method is additive manufacturing; 3D objects are manufactured through the successive deposition of material in layers to achieve a model [15,30]. The second method is subtractive manufacturing; 3D objects are manufactured using the successive milling of extra material from a solid volume of material according to the digital model [31,32]. In prosthodontics, subtractive manufacturing is commonly associated with CAD/CAM technology and has been extensively used to fabricate partial or complete veneers or/and crowns, both of which are types of removable dentures; implant abutments; and prostheses replacing maxillofacial structures [30,31,32,33,34,35]. Figure 1 shows the workflow of manufacturing CECDs.

CAD/CAM manufacturing of computer-engineered complete dentures is associated with several advantages, such as fewer clinical visits with reduced chair time. The denture itself has superior strength and uniform thickness and proper fitting. The digital nature of the system means that they are easily reproducible with less time-consuming tooth setups, easy data backup, and the ease of construction of duplicate prostheses [33]. However, CECDs have some drawbacks. It is difficult to assess proper occlusal vertical dimensions (OVDs), the incisal edge position of the maxillary anterior teeth, and appropriate lip support. CAD/CAM dentures are more expensive and require dimensionally stable and temperature-resistant scanning [11,31,33].

Regardless of the prosthesis design and manufacturing method, patient selection is a critical point to be considered when creating a treatment plan. The candidate or subject should be well informed. An adequate bulk of alveolar bone and an even maxilla–mandibular occlusal relationship (avoiding Angle class 2) allow for better prognostic cases. Edentulous patients with non-aesthetic demands and who do not have TM joint problems can also be selected [34].

Currently, six systems are available for the fabrication of CAE-CDs: the 3Shape Dental System, AvaDent Digital Dentures, Dentca Digital Denture, Wieland Digital Denture, and the Ceramill Full Denture System, the Baltic Denture System, and the VITA VIONIC Digital System [5,6,7,31,32,33,34,35,36]. The majority of the systems use subtractive manufacturing to make their dentures and only use closed systems.

The Baltic Denture System, and VITA VIONIC material types, on the other hand, have an open framework that allows users to select from a variety of handling procedures. It can be used with a variety of open-ended digital scanners, CAD applications, and milling equipment.

The dentist′s prosthodontic skill, the number of dentures required, and denture individualization requirements may all influence the procedure that is chosen. Techniques and technologies are constantly evolving to overcome or minimize patient displeasure with aesthetics, bulkiness, and retention. In all procedures, post-insertion modifications are made. Old dentures can be used for manufacturing new CECDs. Most systems accept external denture staining. Esthetic, retention, tooth size, vertical dimensions, horizontal relationship, and the patient profile are all improved with virtual denture try-in and are highly recommended. Most techniques use white acrylic resin. The interpretation of the digital preview is challenging. All procedures keep a digital record. This is beneficial for seniors with reduced access to oral care. Cast fabrication and polymerization procedures are eliminated, as is the use of monomers as well as the consequences of using this material [36].

### 2.4. Fabrication of Conventional Complete Dentures

**Figure 2:**
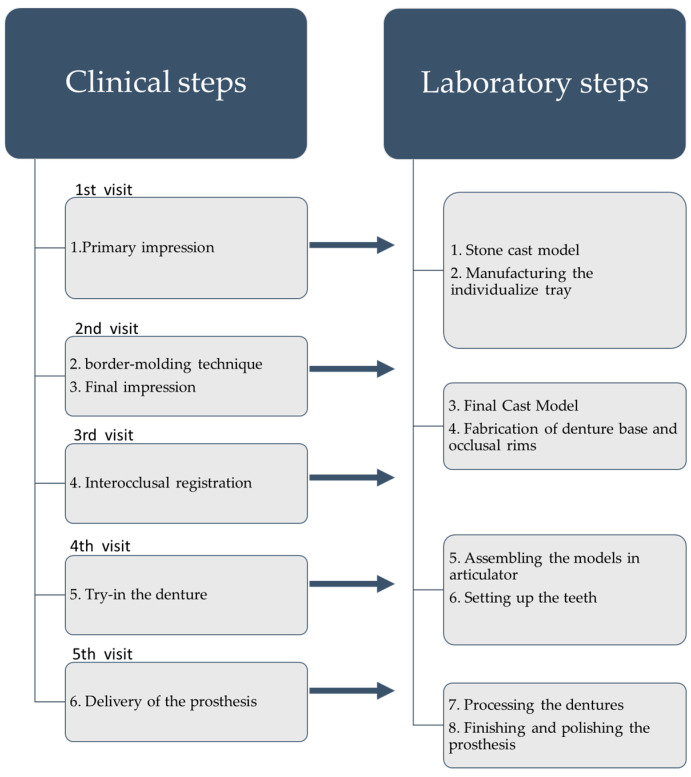
Workflow of conventional CDs.

Fabrication consists of clinical sessions along with the required laboratory sessions and later post-operative adjustment visits. Figure 2 shows the sequence of the clinical and laboratory steps.

## 3. Results

### 3.1. Study Selection and Collections

A total of 102 articles were obtained from the databases using the literature search strategy. A total of eighteen articles were included in this review. Table 1 summarizes the salient details of the studies [10,12,14,15,16,17,18,19,20,21,22,23,24,25].

**Table 1:** Summary of articles where CAD/CAM technologies were used in the fabrication of CECDs in different countries.

Author(s) /Year/ Study Type	Country/Number of Visits	Subjects	Technique	Findings
Mai et al., 2020/Cast Model study [13]	Republic of Korea/ 2 Visits	Edentulous Jaw Models of Maxilla and Mandible	VDFP */CAD-CAM *	Base and dental parts of new dentures were designed efficiently and predictably. Digital protocol facilitates the design process, border seal, and tooth arrangement.
Srinivasan, 2019 /Case report [37]	Switzerland/ 3 Visits	Male/65 years/Maxillary and Mandibular CD *	VDFP */Anatomic Measuring Device (AMD *) AvaDent/CAD-CAM *	Production of clinically acceptable CECDs reduced the number of clinical visits without the use of complex equipment.
		Male/71 years/Maxillary and Mandibular Resin RPD		
Lee et al., 2019/ Case report [15]	Korea/ 2 Visits	Male/53 years/Maxillary and Mandibular CD *	VDFP */CAD-CAM *	Addition of conventional impression and maxillomandibular relationship with laboratory steps using CAD-CAM * technology. Minimized the clinical time.
Goodacre et al., 2018/Case report [38]	USA/ 2 Visits	Male/78 years/Maxillary CD * and Mandibular Overdenture by Dental Implants	VDFP */Anatomic Measuring Device (AMD *) AvaDent/CAD-CAM *	Intraoral scanning captured true mucostatic impression, achieving good retention and stability of the CECD prostheses. Digitally recording tooth location and base morphology of the present dentures reduced the number of clinical steps and eliminated the need to transport conventional impressions to the laboratory.
Contrepois et al., 2018/Case report [16]	France/ 2 Visits	Female/78 years/Maxillary and Mandibular CD *	VDFP */CAD-CAM *	Designing the shape of the teeth for each patient results in better denture customization as well as the appropriate level of tooth staining and an appropriate denture base. Full CECD fabrication ensured a good aesthetic result.
Janeva et al., 2017/Case report [17]	Macedonia/ 3 Visits	Male/63 years/Maxillary and Mandibular CD *	VDFP */Anatomic Measuring Device (AMD *) AvaDent/CAD-CAM *	Combined advantages of CAD/CAM * and traditional clinical recording methods. CAD/CAM * technology eliminated many laboratory steps and simplified the process.
Ohkubo et al., 2017/Case report [18]	Japan/ 2 Visits	Female/82 years/Maxillary and Mandibular CD *	VDFP */DENTCA Piezography Technique CAD-CAM *	Concept of neutral zone and denture space were verified, and denture teeth and flange forms were appropriately designed.
AlHelal et al., 2017/ Case report [39]	Saudi Arabia/ 2 Visits	Male/20-Maxillary CD *	VDFP */CAD-CAM * Monolithic Denture	CECDs minimized the number of appointments, enhanced fitting, and retention, and allowed automated archiving.
Yilmaz et al., 2017/ Cast Model study [12]	Turkey/ 2 Visits	Edentulous Jaw Models of Maxilla and Mandible	VDFP */CAD-CAM *	CECDs do not optimally assess maxillomandibular relationships, maxillary incisal edge placement, and lip support. Creating a mandibular occlusal plane was not possible and resulted in higher costs.
Bajunaid SO /2016/Case report [10]	Saudi Arabia/ 2 Visits	Female/67 years/Maxillary CD * and Mandibular Complete Overdenture	VDFP */CAD-CAM *	Excellent denture base contact, which reduced the number of required dental appointments. Unsatisfactory aesthetic outcomes can be corrected with more experience. Authors recommended the use of this technique in dental school for all levels of study.
de Mendonça et al., 2016/Case report [19]	Brazil/ 3 Visits	Female/63 years/Maxillary CD * and Mandibular Complete Overdenture	VDFP */Prototype then 3D CAD-CAM *	CECDs eliminate acrylic base shrinkage and decreased porosity compared to conventionally processed dentures. CECDs decreased the retention of Candida albicans. Posteriorly, teeth modified and merged into milled sockets with a milled base.
Kim et al., 2016/Case report [20]	Republic Korea/3 Visits	Male/75 years/Maxillary and Mandibular CD *	VDFP */Dentca; CAD/CAM *	Dentures were delivered during 2nd visit with a reduction in the number of clinical and laboratory steps. Clinically acceptable CECDs with smooth surfaces.
		Female/61 years/Maxillary and Mandibular CD *		
Joda et al., 2016/Case report [25]	Switzerland/ 4 Visits	Male/75 years/Maxillary and Mandibular CD * Patient with dental implants	VDFP */Digital Denture Provisional (DDP) CAD-CAM *	Virtually designed and monolithic milled structure. Patient benefits from time and cost savings. Digitalization technique is ideal for planning and provisional steps.
Bilgin et al., 2015/Cast Model study [21]	Turkey/ 3 Visits	Edentulous Jaw Models of Maxilla and Mandible	One-set aligned Artificial tooth System CAD- CAM * and Rapid Prototyping (RP)	CAD/CAM * and RP reduce chair time. Achieved self-designed aesthetics, occlusion, and increased durability. Good for single CD * opposed natural dentition fabricated using RP or CAD/CAM *.
Bidra et al., 2016/Clinical Study [22]	Canada/ 2 Visits	10 Maxillary CD * or Implant-Retained Overdentures	VDFP */CAD-CAM * Monolithic Denture	All dentures in a good state after a 12-months follow-up. Retention loss and excessive tooth wear were observed in five cases. Higher patient satisfaction (79%) regarding their CECDs.
	Canada/ 2 Visits	10 Mandibular Complete or Implant-Retained Overdentures		
Infante et al., 2014/Case report [23]	USA/ 2 Visits	Male/62 years/Maxillary and Mandibular CD *	VDFP */Anatomic Measuring Device (AMD *) AvaDent/CAD-CAM *	Used AMD * clinical records during a one-step appointment. AMD * device allowed the collection of all of the necessary clinical information Virtual denture was milled without the use of stone models and processing.
Kattadiyil et al., 2013/Case report [40]	USA/ 2 Visits	Female/56 years/Maxillary and Mandibular CD *	VDFP */Anatomic Measuring Device (AMD *) AvaDent/CAD-CAM *	Final impressions for both arches, border molding, jaw relationship, and tooth arrangements were made in the first appointment. Less clinical time was required. Polymerization shrinkage was eliminated.
	USA/ 2 Visits	Male/54 year/Maxillary and Mandibular CD *	VDFP */Dentca CAD/CAM *	
Kanazawa et al., 2011/Cast Model study [24]	Japan/ 2 Visits	Edentulous Jaw Models of Maxilla and Mandible	VDFP */CAD-CAM * Monolithic Denture	Dental 3D CBCT * used to process the 3D STL morphological file for the artificial teeth. CD * manufacturing using CAD/CAM * caused large deviations between manufactured teeth and sockets on the prostheses base.

* Abbreviations: virtual design and fabrication process—VDFP; computer-aided design/computer-aided manufacturing—CAD/CAM; computer-aided engineering—CAE; anatomic measuring device—AMD; complete denture—CD; removable partial denture—RPD centric relation—CR; cone beam-computed tomography—CBCT.

### 3.2. Study Characteristics and Quality of the Reports

A majority of the studies were published in First World countries of Europe and North America. Asia is represented by Korea and Japan. Saudi Arabia, Turkey, and Macedonia represent the publishing on CECDs from the Middle East and North Africa region.

Most of the studies were published after 2015 (15 studies: 83%). Twelve were case reports that were published after a 1-year follow-up, one study was a case series [20], and one was a clinical study [22] involving 20 maxillary and mandibular arches with implant-retained prostheses and a 12-month follow-up, and four studies were cast or model evaluations. The following were included in the presented studies: 17 maxillary CDs; 13 mandibular CDs, 4 on cast model; 10 maxillaries with an implant; 11 mandibular overdentures with implants; and 3 mandibular overdentures. Out of the total number, 17 (almost 95%) were VDFP-fabricated using CAD/CAM and the One set aligned Artificial tooth System CAD/CAM and Rapid Prototyping (RP). The maximum number of VDFP CAD/CAM obtained by VDFP with AMD AvaDent/CAD-CAM was eight (44%); two were obtained in the form of a Monolithic Denture and using the DENTCA Piezography Technique and the Digital Denture Provisional technique. The majority of the patients were above 50 years old.

**Figure 3:**
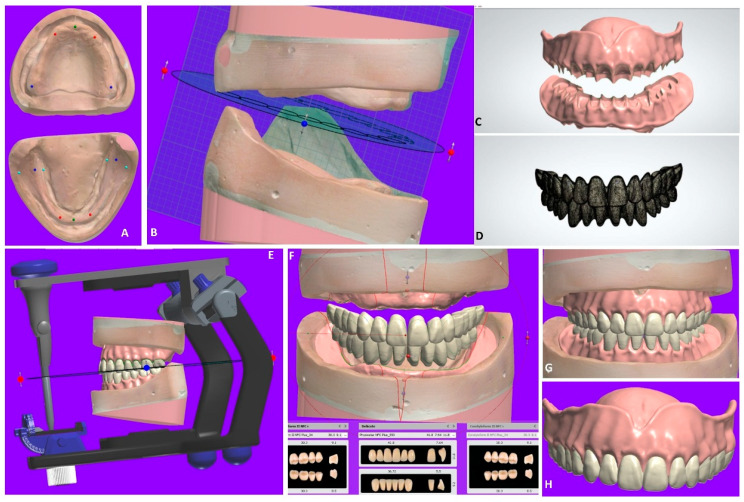
CECDs during digital processing (**A**–**H**). (**A**) Virtual models. (**B**) Interocclusal relationship. (**C**) Denture base planning. (**D**) Tooth setup design. (**E**) Victual articulator. (**F**) Aesthetic parameters and minor corrections. (**G**) Final model. (**H**) Milled prosthesis after fabrication.

Figure 3 summarizes CECD planning and manufacturing according to the workflow presented in the literature. The mounting of the final maxillary and mandibular cast with the occlusal rims can be performed digitally by software that has been connected to CAM. Then, the tooth arrangements are completed with denture teeth (SR Vivodent DCL, Ivoclar Vivadent GmbH) bonded in the milled recesses followed by a clinical try-in for the maxillary and mandibular arch. Finally, computer-engineered complete denture insertion is performed (Figure 3A–H).

During the trial periods for both sets of dentures, the phonetics, aesthetics, and vertical dimensions should be checked. At the time of insertion, the following factors need to be evaluated: retention, stability, occlusion, teeth arrangement, aesthetics, and patient satisfaction.

### 3.3. Synthesis of Results

**Figure 4:**
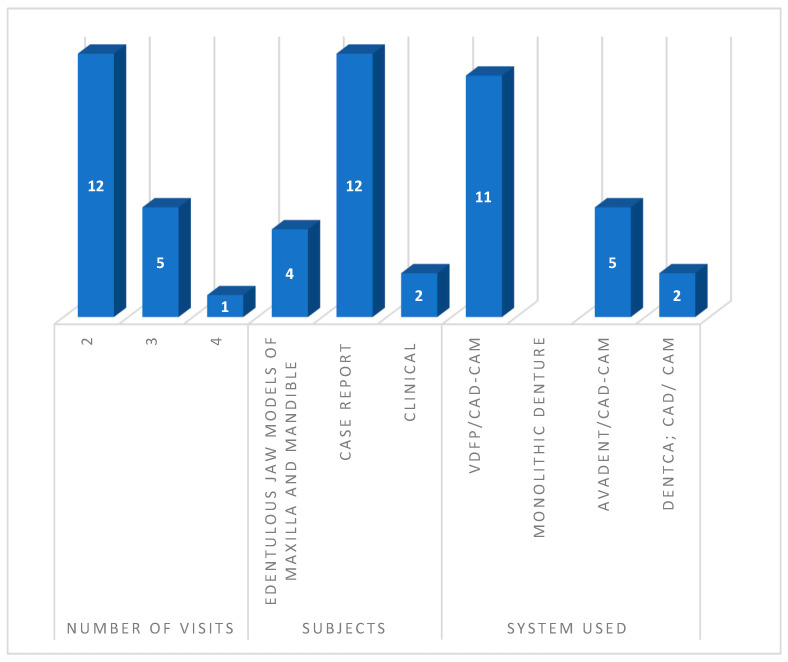
Summary of the study characteristics used in the review.

Few studies suffered from a lack of detail in their reporting i.e., they did not mention gender or report any complications. Details regarding the try-in were not reported. Authors reported problems related to aesthetics, the sizes of the arranged teeth, the position of the teeth with the arches, and the profile of the patient in the summarized studies. In addition to the information in Figure 4, most of the patients were more than 53 years old, and studies from around the world were included. 

## 4. Discussion

The objective of this review was to examine and contrast the fabrication techniques of a computer-engineered complete denture and conventional CD dental prostheses. We examined various relevant parameters such as materials, retention, the accuracy of fitting, aesthetics, fabrication time, patient satisfaction, and the number of post-placement adjustment appointments.

### 4.1. Retention and Fitting

The retention offered by milled pre-polymerized computer-engineered complete denture (CECD) bases with polymethyl methacrylate can be higher than that offered by conventional heat-polymerized denture bases [39]. The CECDs showed a more precise base fit, better clinical retention, and a minimized occurrence of denture-related traumatic lesions [4,31,41,42]. Steinmassl et al. reported that the milled digital removable complete denture demonstrated a significant increase and improvement in retention, fitting [30], and higher dimensional accuracy, contour, fitting, extension, and stability compared to the conventionally fabricated CDs [40,43,44]. However, in a pilot cohort screening, Bidra et al. stated that about 50% of participants did not record retention, adaptation, and stability as having a good or excellent outcome [22]. In CECDs, the greatest amount of misfit is usually on the intaglio surface in the posterior palatal and the border seal areas [30]. These dentures need to be rebased after long periods of use to improve retention and to compensate for physiological bone resorption.

### 4.2. Denture Surface Quality

The CAD-CAM monolithic removable prostheses produced the best combination of precision and duplicability [38]. A smooth surface is important in every restorative treatment to reduce biofilm formation. It lends better esthetics, patient acceptance, and clinical success [45]. Al Moaleem et al. [46] concluded that the presence of smooth restoration, as well as oral prostheses, clinically minimizes adherence and biofilm formation.

Altering the denture-fitting surfaces characteristics, such as porosity and surface irregularity, is essential for reducing *Candida* adhesion on the polymer surface. Denture stomatitis (DS) is an inflammatory condition affecting the palatal mucosa beneath dentures. *Candida* plays a role in palatal inflammation due to accumulation and colony formation, establishing a biofilm [47]. The smooth surface characteristics of CECDs offered less purchase for *Candida* adherence [48,49]. CECDs had lower porosity and polymerization shrinkage. Even after proper finishing and polishing, conventional CDs were associated with increased *Candida* adhesion compared to the CAD/CAM polymers [50].

### 4.3. Clinical Time

Computer-engineered complete dentures required fewer post-insertion adjustments. Saponaro et al. reported that 6 out of 48 contributors did not require any post-insertion adjustment appointment [51]; 16 of the patients only required one post-insertion visit; less than 16 participants required two visits, and 25% (12) of applicants required three or more visits. Bidra et al. found that 3.3% of dentures required modifications after 12 months for all patients [22]. CECDs can be an effective and time-efficient option for completely edentulous patients in terms of reduced time and overall results [40]. Finally, 77% of the patients with edentulism agreed that their new CECDs were “better” than their previous set of CDs [52].

CECDs are a viable alternative to conventional CDs based on the treatment duration and the reduced number of clinical and follow-up appointments, adjustments, and maintenance required [11]. In conventional complete dentures, the follow-up starts immediately after the first 24 h, and as many as three adjustment visits are required [53].

### 4.4. Patient Satisfaction

Participants who received computer-engineered complete dentures showed high levels of satisfaction [22,40,52]. Patient outcomes were clinically acceptable. The retention with milled pre-polymerized denture bases was comparable to that of conventional denture bases [39]. Inokoshi et al. observed that patients were equally satisfied with digital and conventional dentures [54].

As the clinical try-in step is not performed in CECDs, it is not possible to perform an aesthetic or phonetic evaluation, which can lead to better patient satisfaction later on. In conventional CDs, there is a try-in stage (in wax) that makes it possible to change the tooth set-up, meaning that adjustments can be made to customize the denture based on the facial characteristics of each patient.

### 4.5. Manufacturing Time

The digital method was associated with reduced fabrication time and higher technique accuracy [40,41,54]. However, Schwindling and Stober [55] and Wulfman et al. [56] dissented from this characterization and reported a longer working time using the digital procedure. CAD/CAM technology simplifies the laboratory effort, allowing the dental technician to conveniently construct precise and well-organized prostheses [11,57].

### 4.6. Materials Selection

Polymethyl methacrylate (PMMA) is the most widely used material for complete denture fabrication due to its aesthetics, low water absorption and solubility, adequate strength, ease of maintenance, and simple manufacturing process [58]. However, the material has a few disadvantages namely porosity, residual monomer, possible allergens, increased finishing time, brittleness, and uneven thickness [59]. PMMA is also used in computer-engineered complete dentures, which can be pre-polymerized, cross-linked, or high-impact resistant. When used with CAD/CAM, this material shows reduced residual monomer, superior fit, and strength [60].

### 4.7. Complementary Aspects

Computer-engineered complete dentures (CECDs) are superior to the rapidly prototyped conventional dentures in terms of the trueness of the intaglio surfaces [37,56,61]. Bacali et al. stated that it was possible to achieve improved speed, precision, data reproducibility, comfort, chewing efficiency, and reduced costs due to the standardization of the treatment steps in CECDs [62]. Any limitations and disadvantages could be overcome once the digital workflow became familiar [16].

CECDs are slightly lacking in terms of aesthetics, since no try-in steps are involved in their manufacture [42]. Alhallak and Nankali stated that the biocompatibility of CECDs still requires better follow-up and documentation [63]. Data storage allows for a quick replacement of dentures when they are missing or damaged [43,44,56]. Additionally, the time needed for the manufacturing and processing of CECDs was only two visits, resulting in one less hour of chair time for the dentist and five hours less time for the dental laboratory. Ultimately, Peroz et al. concluded that positive changes in the oral health-related quality of life were observed in the participants [64].

The production of CECDs using 3D printing technology is becoming more popular in dental centers. Nevertheless, evidence regarding biocompatibility, the clinical or long-term follow-up of the patients, the chewing load capability [65], and data on the clinical performance of 3D printed dentures are still lacking [66]. Further studies are essential to elucidate these parameters.

Variables related to occlusal forces were examined between CECDs and conventional methods employing various occlusal schemes. CECDs showed better retention of occlusal schemes. Bilateral balanced and lingualized occlusal schemes provided better centralization of forces, improved distribution, and high maximum occlusal forces [67]. This is important, as a significant relationship exists between the distribution of occlusal contacts and temporomandibular joint disorders (TMD) [68]. Any asymmetry in the occlusal contact pattern may precipitate TMD over time.

A remarkable feature of CECDs is the ease of fabrication and reduced chair time required. This is of particular relevance in light of the ongoing COVID-19 pandemic. CECDs would reduce the risk of virus transmission as well as minimize contacts and droplet-generating procedures. This is advantageous for patients, clinicians, and auxiliary staff. The digital storage of patient data would allow for the fabrication of a denture even without the patient visiting a prosthodontist, reducing the risk to older, vulnerable patients [69]. Using the chlorhexidine mouthwash as an antiseptic therapy can have clinical and microbiological benefits [70]. Chlorhexidine gels cause limited changes to the color and mechanical properties of PMMA denture bases. Newer formulations contain anti-discoloring agents that can be safely used with CECDs for maintaining oral hygiene.

## 5. Conclusions

Based on the available literature, it is clear that computer-engineered complete dentures made using CAD/CAM with a digital workflow have several advantages over conventional dentures. The digital workflow can reduce clinical and laboratory time. The patient data stored are invaluable during future appointments. Meticulous care must be taken at each stage from the initial impression to milling to minimize processing errors. Further randomized clinical trials are essential to extensively cover all of the parameters used in computer-engineered complete dentures manufacturing. This paper will aid in the decision-making process during treatment planning for oral healthcare providers.

## Data Availability

Data are available upon request.
